# An extension of the mixed integer part of a nonlinear form

**DOI:** 10.1186/s13660-017-1440-x

**Published:** 2017-07-19

**Authors:** Yunhan Wang, Jiani Mu

**Affiliations:** 1 Department of Computer Science and Technology, Harbin Engineering University, Harbin, 150001 China; 2 Faculty of Science and Technology, Tapee College, Surathani, 84000 Thailand

**Keywords:** diophantine approximation, mixed power, integer variables

## Abstract

Our aim in this paper is to consider the integer part of a nonlinear form representing primes. We establish that if $\lambda_{1},\lambda _{2},\ldots,\lambda_{8}$ are positive real numbers, at least one of the ratios $\lambda_{i}/\lambda_{j}$ ($1\leq i< j\leq 8$) is irrational, then the integer parts of $\lambda_{1}x_{1}^{3}+\lambda_{2}x_{2}^{3}+ \lambda_{3}x_{3}^{4}+\lambda_{4}x_{4}^{4}+\lambda_{5}x_{5}^{4} + \lambda_{6}x_{6}^{5}+\lambda_{7}x_{7}^{5}+\lambda_{8}x_{8}^{5}$ are prime infinitely often for $x_{1},x_{2},\ldots,x_{8}$, where $x_{1},x_{2},\ldots,x_{8}$ are natural numbers.

## Introduction

The integer part of linear and nonlinear forms representing primes has been considered by many scholars. Let $[x]$ be the greatest integer not exceeding *x*. In 1966, Danicic [1] proved that if the diophantine inequality

1 $$ \vert \lambda_{1}p_{1}+\lambda_{2}p_{2}+ \lambda_{3}p_{3}+\eta \vert < \varepsilon $$

satisfies certain conditions, and primes $p_{i}\leq N$ ($i=1,2,3$), then the number of prime solutions $(p_{1},p_{2},p_{3},p_{4})$ of (1) is greater than $CN^{3}(\log N)^{-4}$, where *C* is a positive number independent of *N*. Based on the above result, Danicic [1] proved that if *λ*, *μ* are non-zero real numbers, not both negative, *λ* is irrational, and *m* is a positive integer, then there exist infinitely many primes *p* and pairs of primes $p_{1}$, $p_{2}$ and $p_{3}$ such that

$$[\lambda p_{1}+\mu p_{2}+\mu p_{3}]=mp. $$

In particular, $[\lambda p_{1}+\mu p_{2}+\mu p_{3}]$ represents infinitely many primes.

Brüdern et al. [2] proved that if $\lambda_{1},\ldots, \lambda_{s}$ are positive real numbers, $\lambda_{1}/\lambda_{2}$ is irrational, all Dirichlet L-functions satisfy the Riemann hypothesis, $s\geq \frac{8}{3}k+2$, then the integer parts of

$$\lambda_{1}x^{k}_{1}+\lambda_{2}x^{k}_{2}+ \cdots +\lambda_{s}x^{k} _{s} $$

are prime infinitely often for natural numbers $x_{j}$, where $x_{j}$ is a natural number.

Recently, Lai [3] proved that for integer $k\geq 4$, $r\geq 2^{k-1}+1$, under certain conditions, there exist infinitely many primes $p_{1},\ldots,p_{r},p$ such that

1.1 $$ \bigl[\mu_{1} p_{1}^{k}+\cdots + \mu_{r} p_{r}^{k}\bigr]=mp. $$

It is natural to ask if the above results are true when primes $p_{j}$ in (1.1) are replaced by natural numbers $x_{j}$. In this paper we shall give an affirmative answer to this question.

## Main result

Our main aim is to investigate the integer part of a nonlinear form with integer variables and mixed powers 3, 4 and 5. Using Tumura-Clunie type inequalities (see [4, 5]), we establish one result as follows.

### Theorem 2.1

*Let* $\lambda_{1},\lambda_{2},\ldots,\lambda_{8}$ *be nonnegative real numbers*, *at least one of the ratios* $\lambda_{i}/ \lambda_{j}$ ($1\leq i< j\leq 8$) *is rational*. *Then the integer parts of*

$$\lambda_{1}x_{1}^{3}+\lambda_{2}x_{2}^{3}+ \lambda_{3}x_{3}^{4}+\lambda _{4}x_{4}^{5}+ \lambda_{5}x_{5}^{6} +\lambda_{6}x_{6}^{7}+ \lambda_{7}x _{7}^{8}+\lambda_{8}x_{8}^{5} $$

*are prime infinitely often for* $x_{1},x_{2},\ldots,x_{8}$, *where* $x_{1},x_{2},\ldots,x_{8}$ *are natural numbers*.

### Remark

It is easy to see from Theorem 2.1 that primes $p_{j}$ in (1.1) are replaced by natural numbers $x_{j}$ and there exist infinitely many primes $p_{1},\ldots ,p_{r}$ and *p* such that $[\mu_{1} p_{1}^{k}+\cdots +\mu_{r+1} p_{r+1}^{k}]=mp_{r}$, where *m* is a nonnegative integer (see [6]).

## Outline of the proof

Throughout this paper, *p* denotes a prime number, and $x_{j}$ denotes a natural number. *δ* is a sufficiently small positive number, *ε* is an arbitrarily small positive number. Constants, both explicit and implicit, in Landau or Vinogradov symbols may depend on $\lambda_{1},\lambda_{2},\ldots,\lambda_{8}$. We write $e(x)=\exp (2 \pi i x)$. We take *X* to be the basic parameter, a large real integer. Since at least one of the ratios $\lambda_{i}/\lambda_{j}$ ($1\leq i< j \leq 8$) is irrational, without loss of generality, we may assume that $\lambda_{1}/ \lambda_{2}$ is irrational. For the other cases, the only difference is in the following intermediate region, and we may deal with the same method in Section 4.

Since $\lambda_{1}/ \lambda_{2}$ is irrational, there are infinitely many pairs of integers *q*, *a* with $\vert \lambda_{1}/\lambda_{2}-a/q\vert \geq {q^{-1}}$, $(p,q)=2$, $q>0$ and $a\neq 0$. We choose *p* to be large in terms of $\lambda_{1},\lambda_{2},\ldots,\lambda_{8}$, and make the following definitions.

Put $\tau =N^{-1+\delta }$,$T=N^{\frac{2}{5}}$, $L=\log N$, $Q=(\vert \lambda_{1}\vert ^{-2}+\vert \lambda_{2}\vert ^{-3})N^{2-\delta }$, $[N^{1-3 \delta }]=p$ and $P=N^{3\delta }$, where $N\asymp X$. Let *ν* be a positive real number, we define

3.1 $$\begin{aligned}& \begin{aligned}&K_{\nu }(\alpha)=\nu \biggl(\frac{\sin \pi \nu \alpha }{\pi \nu \alpha }\biggr)^{2},\quad \alpha \neq 0,\qquad K_{\nu }(0)=\nu, \\ &F_{i}(\alpha)=\sum_{1\leq x\leq X^{\frac{1}{5}}}e\bigl(\alpha x ^{4}\bigr),\quad i=1,2, \qquad F_{j}(\alpha)=\sum _{1\leq x\leq X^{\frac{1}{6}}}e\bigl(\alpha x^{2}\bigr),\quad j=3,4,5, \\ &F_{k}(\alpha)=\sum_{1\leq x\leq X^{\frac{1}{7}}}e\bigl(\alpha x ^{3}\bigr),\quad k=6,7,8, \qquad G(\alpha)=\sum_{p\leq N}( \log p)e(\alpha p), \\ &f_{i}(\alpha)= \int_{1}^{X^{\frac{1}{4}}}e\bigl(\alpha x^{5}\bigr)\,dx,\quad i=1,2,\qquad f_{j}(\alpha)= \int_{1}^{X^{\frac{1}{5}}}e\bigl(\alpha x^{6}\bigr)\,dx,\quad j=3,4,5, \\ &f_{k}(\alpha)= \int_{1}^{X^{\frac{1}{5}}}e\bigl(\alpha x^{4}\bigr)\,dx,\quad k=6,7,8, \qquad g(\alpha)= \int_{2}^{N}e(\alpha x)\,dx. \end{aligned} \end{aligned}$$

From (3.1) we have

$$\begin{aligned} J =:& \int_{-\infty }^{+\infty }\prod_{i=1}^{9}F_{i}( \lambda _{i}\alpha) G(-\alpha)e\biggl(-\frac{1}{2}\alpha \biggr)K_{\frac{1}{2}}(\alpha)\,d\alpha \\ \leq & \log N\sum_{{\vert \lambda _{1}x_{1}^{3}+\lambda _{2}x_{2}^{3}+\lambda _{3}x_{3}^{4}+\lambda _{4}x_{4}^{4} +\lambda _{5}x_{5}^{5}+\cdots +\lambda _{9}x_{9}^{5}-p-\frac{1}{2}\vert < \frac{1}{4}\atop {1\leq x_{1},x_{2}\leq X^{1/5}, 1\leq x_{3},x_{4}\leq X^{1/4},1\leq x_{5},\ldots,x_{9}\leq X^{1/6}, p\leq N}}}1, \end{aligned}$$

which gives that

$${\mathcal{N}}(X)\geq (\log N)^{-3}J^{2}. $$

Next comes the time to estimate *J*. As usual, we split the range of infinite integration into three sections, $\frak{C}=\{\alpha \in {\mathbb{R}}:\vert \alpha \vert \leq \tau \}$, $\frak{D}=\{\alpha \in {\mathbb{R}}:\tau <\vert \alpha \vert \leq P\}$, $\frak{c}=\{\alpha \in {\mathbb{R}}:\vert \alpha \vert >P\}$ named the neighborhood of the origin, the intermediate region, and the trivial region, respectively.

In Sections 3, 4 and 5, we shall establish that $J({\frak{C}})\gg X ^{\frac{121}{60}}$, $J({\frak{D}})=o(X^{\frac{121}{60}})$, and $J({\frak{c}})=o(X^{\frac{121}{60}})$. Thus

$$J\gg X^{\frac{121}{60}},\qquad {\mathcal{N}}(X)\gg X^{\frac{121}{60}}L ^{-1}, $$

namely, under the conditions of Theorem 1.1,

3.2 $$ \biggl\vert \lambda_{1}x_{1}^{3}+ \lambda_{2}x_{2}^{3}+\lambda_{3}x_{3}^{4}+ \lambda_{4}x_{4}^{5}+\lambda_{5}x_{5}^{6} +\lambda_{6}x_{6}^{7}+ \lambda_{7}x_{7}^{8}+ \lambda_{8}x_{8}^{5}-p-\frac{1}{2}\biggr\vert < \frac{1}{2} $$

has infinitely many solutions in positive integers $x_{1},x_{2}, \ldots,x_{8}$ and prime *p*. From (3.2) we have

$$p< \lambda_{1}x_{1}^{3}+\lambda_{2}x_{2}^{3}+ \lambda_{3}x_{3}^{4}+ \lambda_{4}x_{4}^{5}+ \lambda_{5}x_{5}^{6} +\lambda_{6}x_{6}^{7}+ \lambda_{7}x_{7}^{8}+\lambda_{8}x_{8}^{5}< p+2, $$

which gives that

$$\bigl[\lambda_{1}x_{1}^{3}+\lambda_{2}x_{2}^{3}+ \lambda_{3}x_{3}^{4}+ \lambda_{4}x_{4}^{5}+ \lambda_{5}x_{5}^{6} +\lambda_{6}x_{6}^{7}+ \lambda_{7}x_{7}^{8}+\lambda_{8}x_{8}^{5} \bigr]=p. $$

The proof of Theorem 1.1 is complete.

## The neighborhood of the origin

### Lemma 4.1

see [7], Theorem 4.1

*Let* $(a,q)=1$. *If* $\alpha =a/q+\beta $, *then we have*

$$\sum_{1\leq x\leq N^{1/t}}e\bigl(\alpha x^{t} \bigr)=q^{-1}\sum_{m=1}^{q}e \bigl(am^{t}/q\bigr) \int_{1}^{N^{1/t}}e\bigl(\beta y^{t}\bigr)\,dy+O \bigl(q^{1/2+ \varepsilon }\bigl(1+N\vert \beta \vert \bigr)\bigr). $$

Lemma 4.1 immediately gives that

4.1 $$ F_{i}(\alpha)=f_{i}(\alpha)+O\bigl(X^{\delta } \bigr), $$

where $\vert \alpha \vert \in \frak{C}$ and $i=1,2,\ldots,8$.

### Lemma 4.2

see [8], Lemma 3 and Remark 2

*Let*

$$\begin{aligned}& I(\alpha)=\sum_{\vert \gamma \vert \leq T, \beta \geq \frac{2}{3}} \sum _{n\leq N}n^{\rho -1}e(n\alpha), \\& J(\alpha)=O \bigl( \bigl(1+\vert \alpha \vert N\bigr)N^{\frac{2}{3}}L^{C} \bigr), \end{aligned}$$

*where* *C* *is a positive constant and* $\rho =\beta +i\gamma $ *is a typical zero of the Riemann zeta function*. *Then we have*

$$\begin{aligned}& \int_{-\frac{1}{2}}^{\frac{1}{2}}\bigl\vert I(\alpha)\bigr\vert ^{2}\,d\alpha \ll N\exp \bigl(-L ^{\frac{1}{5}}\bigr), \\& \int_{-\tau }^{\tau }\bigl\vert J(\alpha)\bigr\vert ^{2}\,d\alpha \ll N\exp \bigl(-L^{ \frac{1}{5}}\bigr) \end{aligned}$$

*and*

$$G(\alpha)=g(\alpha)-I(\alpha)+J(\alpha). $$

### Lemma 4.3

see [8], Lemma 5

*For* $i=1,2$, $j=3,4,5$, $k=6,7,8$, *we have*

$$\int_{-\frac{1}{2}}^{\frac{1}{2}}\bigl\vert f_{i}(\alpha) \bigr\vert ^{2}\,d\alpha \ll X ^{-\frac{1}{3}},\qquad \int_{-\frac{1}{2}}^{\frac{1}{2}}\bigl\vert f_{j}(\alpha) \bigr\vert ^{2}\,d\alpha \ll X^{-\frac{1}{2}},\qquad \int_{-\frac{1}{2}}^{ \frac{1}{2}}\bigl\vert f_{k}(\alpha) \bigr\vert ^{2}\,d\alpha \ll X^{-\frac{3}{5}}. $$

### Lemma 4.4

*We have*

$$\int_{{\frak{C}}}K_{\frac{1}{2}}(\alpha) \Biggl\vert \prod _{i=1}^{9}F_{i}( \lambda_{i}\alpha)G(-\alpha)-\prod_{i=1}^{9}f_{i}( \lambda_{i}\alpha)g(-\alpha)\Biggr\vert \,d\alpha \ll \frac{X^{\frac{121}{60}}}{L}. $$

### Proof

It is obvious that

$$\begin{aligned}& F_{i}(\lambda_{i}\alpha)\ll X^{\frac{1}{3}},\qquad f_{i}(\lambda_{i} \alpha)\ll X^{\frac{1}{3}},\qquad F_{j}(\lambda_{j}\alpha)\ll X^{ \frac{1}{4}},\qquad f_{j}(\lambda_{j}\alpha)\ll X^{\frac{1}{4}}, \\& F_{k}(\lambda_{k}\alpha)\ll X^{\frac{1}{5}},\qquad f_{k}(\lambda_{k} \alpha)\ll X^{\frac{1}{5}},\qquad G(-\alpha) \ll N \quad \mbox{and}\quad g(-\alpha)\ll N \end{aligned}$$

hold for $i=1,2$, $j=3,4,5$ and $k=6,7,8$.

By (4.1), Lemmas 4.2 and 4.3, we have

$$\int_{{\frak{C}}}\Biggl\vert \bigl(F_{1}( \lambda_{1}\alpha)-f_{1}(\lambda_{1}\alpha) \bigr)\prod_{i=2}^{9} F_{i}( \lambda_{i}\alpha)G(-\alpha)\Biggr\vert K_{ \frac{1}{2}}(\alpha)\,d\alpha \ll \frac{X^{\delta }X^{\frac{101}{60}}N}{N ^{1-\delta }}\ll X^{\frac{101}{60}+2\delta } $$

and

$$\begin{aligned}& \int_{{\frak{C}}}K_{\frac{1}{2}}(\alpha)\Biggl\vert \prod _{i=1}^{9}f_{i}(\lambda_{i} \alpha) \bigl(G(-\alpha)-g(-\alpha)\bigr)\Biggr\vert \,d\alpha \\& \quad \ll X^{\frac{101}{60}} \biggl( \int_{{\frak{C}}}\bigl\vert f_{1}(\lambda_{1} \alpha)\bigr\vert ^{2}K_{\frac{1}{2}}(\alpha)\,d\alpha \biggr)^{\frac{1}{2}} \biggl( \int_{{\frak{C}}}\bigl\vert J(-\alpha)-I(-\alpha)\bigr\vert ^{2}K_{\frac{1}{2}}(\alpha)\,d\alpha \biggr)^{ \frac{1}{2}} \\& \quad \ll X^{\frac{101}{60}} \biggl( \int_{-\frac{1}{2}}^{\frac{1}{2}}\bigl\vert f_{1}( \lambda_{1}\alpha)\bigr\vert ^{2}\,d\alpha \biggr)^{\frac{1}{2}} \biggl( \int_{{\frak{C}}}\bigl\vert J(\alpha)\bigr\vert ^{2}\,d\alpha + \int_{-\frac{1}{2}}^{\frac{1}{2}}\bigl\vert I(\alpha)\bigr\vert ^{2}\,d\alpha \biggr)^{\frac{1}{2}} \\& \quad \ll \frac{X^{\frac{121}{60}}}{L}. \end{aligned}$$

The proofs of the other cases are similar, so we complete the proof of Lemma 4.4. □

### Lemma 4.5

*The following inequality holds*.

$$\int_{\vert \alpha \vert >\frac{1}{N^{1-\delta }}}K_{\frac{1}{2}}(\alpha) \Biggl\vert \prod _{i=1}^{9}f_{i}(\lambda_{i} \alpha)g(-\alpha)\Biggr\vert \,d\alpha \ll X^{\frac{121}{60}-\frac{121}{60}\delta }. $$

### Proof

For $\alpha \neq 0$, $i=1,2$, $j=3,4,5$, $k=6,7,8$, we know that

$$f_{i}(\lambda_{i}\alpha)\ll \vert \alpha \vert ^{-\frac{1}{3}},\qquad f_{j}( \lambda_{j}\alpha)\ll \vert \alpha \vert ^{-\frac{1}{4}},\qquad f_{k}(\lambda _{k}\alpha)\ll \vert \alpha \vert ^{-\frac{1}{5}},\qquad g(-\alpha)\ll \vert \alpha \vert ^{-1}. $$

Thus

$$\int_{\vert \alpha \vert >\frac{1}{N^{1-\delta }}}\Biggl\vert \prod_{i=1}^{9}f_{i}( \lambda_{i}\alpha)g(-\alpha)\Biggr\vert K_{\frac{1}{2}}(\alpha)\,d\alpha \ll \int_{\vert \alpha \vert >\frac{1}{N^{1-\delta }}}\vert \alpha \vert ^{-\frac{181}{60}}\,d\alpha \ll X^{\frac{121}{60}-\frac{121}{60}\delta }. $$

 □

### Lemma 4.6

*The following inequality holds*.

$$\int_{-\infty }^{+\infty }\prod_{i=1}^{9}f_{i}( \lambda_{i} \alpha) g(-\alpha)e\biggl(-\frac{1}{2}\alpha \biggr)K_{\frac{1}{2}}(\alpha)\,d\alpha \gg X^{\frac{121}{60}}. $$

### Proof

We have

$$\begin{aligned}& \int_{-\infty }^{+\infty }\prod_{i=1}^{9}f_{i}( \lambda_{i} \alpha) g(-\alpha)e\biggl(-\frac{1}{2}\alpha \biggr)K_{\frac{1}{2}}(\alpha)\,d\alpha \\& \quad = \int_{1}^{X^{\frac{1}{3}}} \int_{1}^{X^{\frac{1}{3}}} \int_{1}^{X ^{\frac{1}{4}}} \int_{1}^{X^{\frac{1}{4}}} \int_{1}^{X^{\frac{1}{4}}} \int_{1}^{X^{\frac{1}{5}}} \int_{1}^{X^{\frac{1}{5}}} \int_{1}^{X^{ \frac{1}{5}}} \int_{1}^{N} \int_{-\infty }^{+\infty }e\biggl(\alpha \biggl(\lambda _{1}x_{1}^{3}+\lambda_{2}x_{2}^{3}+ \lambda_{3}x_{3}^{4} \\& \qquad {} +\lambda_{4}x_{4}^{4}+\lambda_{5}x_{5}^{4}+ \lambda_{6}x_{6}^{5}+ \lambda_{7}x_{7}^{5}+ \lambda_{8}x_{8}^{5}-x-\frac{1}{2}\biggr) \biggr) \\& \qquad {}\times K_{ \frac{1}{2}}(\alpha)\,d\alpha \,dx \,dx_{8}\,dx_{7}\,dx_{6}\,dx_{5}\,dx_{4}\,dx_{3}\,dx _{2}\,dx_{1} \\& \quad = \frac{1}{72{,}000} \int_{1}^{X}\cdots \int_{1}^{X} \int_{1}^{N} \int_{-\infty }^{+\infty }x_{1}^{-\frac{2}{3}}x_{2}^{-\frac{2}{3}}x _{3}^{-\frac{3}{4}} x_{4}^{-\frac{3}{4}}x_{5}^{-\frac{3}{4}}x_{6}^{- \frac{4}{5}}x_{7}^{-\frac{4}{5}} x_{8}^{-\frac{4}{5}} \\& \qquad {}\times e\Biggl(\alpha \Biggl( \sum _{i=1}^{8}\lambda_{i} x_{i}-x- \frac{1}{2}\Biggr)\Biggr) K_{\frac{1}{2}}(\alpha)\,d\alpha \,dx \,dx_{9}\cdots \,dx_{1} \\& \quad = \frac{1}{72{,}000} \int_{1}^{X}\cdots \int_{1}^{X} \int_{1}^{N}x_{1} ^{-\frac{2}{3}}x_{2}^{-\frac{2}{3}}x_{3}^{-\frac{3}{4}} x_{4}^{- \frac{3}{4}}x_{5}^{-\frac{3}{4}}x_{6}^{-\frac{4}{5}}x_{7}^{- \frac{4}{5}} x_{8}^{-\frac{4}{5}} \\& \qquad {}\times \max \Biggl(0,\frac{1}{2}-\Biggl\vert \sum _{i=1}^{8}\lambda_{i} x_{i}-x- \frac{1}{2}\Biggr\vert \Biggr)\,dx \,dx_{8}\cdots \,dx_{1} \end{aligned}$$

from (2.3).

Let ${\vert \sum_{i=1}^{8}\lambda_{i} x_{i}-x-\frac{1}{2}\vert \leq \frac{1}{2}}$. Then we have

$$\sum_{i=1}^{8}\lambda_{i} x_{i}-\frac{3}{4}\leq x\leq \sum_{i=1}^{8} \lambda_{i} x_{i}-\frac{1}{4}. $$

By using

$$\sum_{i=1}^{8}\lambda_{i} x_{i}-\frac{3}{4}>1 \quad \mbox{and}\quad \sum _{i=1}^{8}\lambda_{i} x_{i}- \frac{1}{4}< N, $$

we obtain that

$$\lambda_{j}X\Biggl(8\sum_{i=1}^{8} \lambda_{i}\Biggr)^{-1} \leq x_{j} \leq \lambda_{j}X\Biggl(4\sum_{i=1}^{8} \lambda_{i}\Biggr)^{-1},\quad j=1, \ldots,8, $$

and hence

$$\begin{aligned}& \int_{-\infty }^{+\infty }\prod_{i=1}^{9}f_{i}( \lambda_{i} \alpha) g(-\alpha)e\biggl(-\frac{1}{2}\alpha \biggr)K_{\frac{1}{2}}(\alpha)\,d\alpha \\& \quad \geq \frac{1}{576{,}000}\prod _{j=1}^{8}\lambda_{j} \Biggl(8 \sum _{i=1}^{8}\lambda_{i} \Biggr)^{-8}X^{\frac{121}{60}}. \end{aligned}$$

Then we complete the proof of this lemma. □

## The intermediate region

### Lemma 5.1

*We have*

$$\begin{aligned}& \int_{-\infty }^{+\infty }\bigl\vert F_{i}( \lambda_{i}\alpha)\bigr\vert ^{8}K_{ \frac{1}{2}}(\alpha)\,d\alpha \ll X^{\frac{5}{3}+\frac{1}{3}\varepsilon }, \\& \int_{-\infty }^{+\infty }\bigl\vert F_{j}( \lambda_{j}\alpha)\bigr\vert ^{16}K_{ \frac{1}{2}}(\alpha)\,d\alpha \ll X^{3+\frac{1}{4}\varepsilon }, \\& \int_{-\infty }^{+\infty }\bigl\vert F_{k}( \lambda_{k}\alpha)\bigr\vert ^{32}K_{ \frac{1}{2}}(\alpha)\,d\alpha \ll X^{\frac{27}{5}+\frac{1}{5}\varepsilon } \end{aligned}$$

*and*

$$\int_{-\infty }^{+\infty }\bigl\vert G(-\alpha)\bigr\vert ^{2}K_{\frac{1}{2}}(\alpha)\,d\alpha \ll NL $$

*for* $i=1,2$, $j=3,4,5$ *and* $k=6,7,8$.

### Proof

We have

$$\begin{aligned}& \int_{-\infty }^{+\infty }\bigl\vert F_{j}( \lambda_{j}\alpha)\bigr\vert ^{16}K_{ \frac{1}{2}}(\alpha)\,d\alpha \\& \quad \ll \sum_{m=-\infty }^{+\infty } \int_{m}^{m+1}\bigl\vert F_{j}( \lambda_{j}\alpha)\bigr\vert ^{16}K_{\frac{1}{2}}(\alpha)\,d\alpha \\& \quad \ll \sum_{m=0}^{1} \int_{m}^{m+1}\bigl\vert F_{j}( \lambda_{j}\alpha)\bigr\vert ^{16}\,d\alpha +\sum _{m=2}^{+\infty }m^{-2} \int_{m}^{m+1}\bigl\vert F_{j}( \lambda_{j}\alpha)\bigr\vert ^{16}\,d\alpha \\& \quad \ll X^{3+\frac{1}{4}\varepsilon } \end{aligned}$$

from (3.1) and Hua’s inequality.

The proofs of others are similar. So we omit them here. □

### Lemma 5.2

see [7], Lemma 2.4 (Weyl’s inequality)

*Suppose that*

$$\biggl\vert \alpha -\frac{a}{q}\biggr\vert \leq \frac{1}{q^{2}}, $$

$(a,q)=1$ *and*

$$\phi (x)=\alpha x^{k}+\alpha_{1}x^{k-1}+\cdots + \alpha_{k-1}x+\alpha _{k}. $$

*Then we have*

$$\sum_{x=1}^{M}e\bigl(\phi (x)\bigr)\ll M^{1+\varepsilon }\bigl(q^{-1}+M^{-1}+qM ^{-k} \bigr)^{2^{1-k}}. $$

### Lemma 5.3

*For every real number* $\alpha \in \frak{D}$, *we have*

$$W(\alpha)\ll X^{\frac{1}{3}-\frac{1}{4}\delta +\frac{1}{3}\varepsilon }, $$

*where*

$$W(\alpha)=\min \bigl(\bigl\vert G_{1}(\tau_{1}\alpha) \bigr\vert ,\bigl\vert G_{2}(\tau_{2}\alpha)\bigr\vert \bigr). $$

### Proof

For $\alpha \in \frak{D}$ and $i=1,2$, we choose $a_{i}$, $q_{i}$ such that $\vert \lambda_{i}\alpha -a_{i}/q_{i}\vert \leq q_{i} ^{-1}Q^{-1}$ with $(a_{i},q_{i})=1$ and $1\leq q_{i}\leq Q$. We note that $a_{1}a_{2}\neq 0$. If $q_{1},q_{2}\leq P$, then

$$\biggl\vert a_{2}q_{1}\frac{\lambda_{1}}{\lambda_{2}}-a_{1}q_{2} \biggr\vert \leq \biggl\vert \frac{a_{2}/q_{2}}{\lambda_{2}\alpha }q_{1}q_{2} \biggl(\lambda_{1}\alpha -\frac{a_{1}}{q_{1}}\biggr)\biggr\vert + \biggl\vert \frac{a_{1}/q_{1}}{\lambda_{2}\alpha }q_{1}q_{2}\biggl( \lambda_{2}\alpha -\frac{a_{2}}{q_{2}}\biggr)\biggr\vert \ll PQ^{-1}< \frac{1}{2q}. $$

We recall that *q* was chosen as the denominator of a convergent to the continued fraction for $\lambda_{1}/\lambda_{2}$. Thus, by Legendre’s law of best approximation, we have $\vert q'\frac{\lambda_{1}}{\lambda_{2}}-a'\vert >\frac{1}{2q}$ for all integers $a'$, $q'$ with $1\leq q'< q$, thus $\vert a_{2}q_{1}\vert \geq q=[N^{1-8\delta }]$. On the other hand, $\vert a_{2}q_{1}\vert \ll q_{1}q_{2}P \ll N^{18\delta }$, which is a contradiction. And so, for at least one *i*, $P< q_{i}\ll Q$. Hence, by Lemma 5.2, we obtain the desired inequality for $W(\alpha)$. □

### Lemma 5.4

*The following inequality holds*.

$$\int_{\frak{D}}\prod_{i=1}^{9}F_{i}( \lambda_{i}\alpha) G(- \alpha)e\biggl(-\frac{1}{4}\alpha \biggr)K_{\frac{1}{3}}(\alpha)\,d\alpha \ll X ^{\frac{147}{50}-\frac{1}{21}\delta +\varepsilon }. $$

### Proof

We have

$$\begin{aligned}& \int_{{\frak{D}}}\prod_{i=1}^{8} \bigl\vert F_{i}(\lambda_{i}\alpha)G(-\alpha)\bigr\vert K_{\frac{1}{2}}(\alpha)\,d\alpha \\& \quad \ll \max_{\alpha \in {\frak{D}}}\bigl\vert W(\alpha)\bigr\vert ^{\frac{1}{4}} \biggl(\biggl( \int_{-\infty }^{+\infty }\bigl\vert F_{1}( \lambda_{1}\alpha)\bigr\vert ^{8}\biggr)^{ \frac{1}{8}} \biggl( \int_{-\infty }^{+\infty }\bigl\vert F_{2}( \lambda_{2}\alpha)\bigr\vert ^{8}\biggr)^{ \frac{3}{32}} \\& \qquad {} +\biggl( \int_{-\infty }^{+\infty }\bigl\vert F_{1}( \lambda_{1}\alpha)\bigr\vert ^{8}\biggr)^{ \frac{3}{32}} \biggl( \int_{-\infty }^{+\infty }\bigl\vert F_{2}( \lambda_{2}\alpha)\bigr\vert ^{8}\biggr)^{ \frac{1}{8}} \biggr) \\& \qquad {} \times \Biggl(\prod_{j=3}^{5} \int_{-\infty }^{+\infty }\bigl\vert F_{j}( \lambda_{j}\alpha)\bigr\vert ^{16} K_{\frac{1}{2}}( \alpha)\,d\alpha \Biggr)^{ \frac{1}{16}} \Biggl(\prod_{k=6}^{8} \int_{-\infty }^{+\infty }\bigl\vert F_{k}( \lambda_{k}\alpha)\bigr\vert ^{32}K_{\frac{1}{2}}(\alpha)\,d\alpha \Biggr) ^{ \frac{1}{32}} \\& \qquad {} \times \biggl( \int_{-\infty }^{+\infty }\bigl\vert G(-\alpha)\bigr\vert ^{2}K_{\frac{1}{2}}( \alpha)\,d\alpha \biggr)^{\frac{1}{2}} \\& \quad \ll \bigl(X^{\frac{1}{3}-\frac{1}{4}\delta +\frac{1}{3}\varepsilon }\bigr)^{ \frac{1}{4}} \bigl(X^{\frac{5}{3}+\frac{1}{3}\varepsilon } \bigr)^{\frac{7}{32}} \bigl(X^{3+\frac{1}{4}\varepsilon }\bigr)^{\frac{3}{16}} \bigl(X^{\frac{27}{5}+ \frac{1}{5}\varepsilon }\bigr)^{\frac{3}{32}}(N L)^{\frac{1}{2}} \\& \quad \ll X^{\frac{121}{60}-\frac{1}{16}\delta +\varepsilon } \end{aligned}$$

from Lemmas 5.1, 5.3 and Hölder’s inequality. □

## The trivial region

### Lemma 6.1

see [9], Lemma 2

*Let*

$$V(\alpha)=\sum e\bigl(\alpha f(x_{1},\ldots,x_{m})\bigr), $$

*where the summation is over any finite set of values of* $x_{1},\ldots,x_{m}$ *and* *f* *is any real function*. *Then we have*

$$\int_{\vert \alpha \vert >A}\bigl\vert V(\alpha)\bigr\vert ^{3}K_{\nu }(\alpha)\,d\alpha \leq \frac{23}{A} \int_{-\infty }^{\infty }\bigl\vert V(\alpha)\bigr\vert ^{3} K_{\nu }(\alpha)\,d\alpha $$

*for any* $A>4$.

The following inequality holds.

### Lemma 6.2

*We have*

$$\int_{\frak{c}}\prod_{i=1}^{9}F_{i}( \lambda_{i}\alpha) G(- \alpha)e\biggl(-\frac{1}{2}\alpha \biggr)K_{\frac{1}{2}}(\alpha)\,d\alpha \ll X ^{\frac{121}{60}-6\delta +\varepsilon }. $$

### Proof

We have

$$\begin{aligned}& \int_{\frak{c}}\prod_{i=1}^{9}F_{i}( \lambda_{i}\alpha) G(- \alpha)e\biggl(-\frac{1}{4}\alpha \biggr)K_{\frac{1}{4}}(\alpha)\,d\alpha \\& \quad \ll \frac{1}{P} \int_{-\infty }^{+\infty }\Biggl\vert \prod _{i=1}^{9}F_{i}(\lambda_{i} \alpha)G(-\alpha)\Biggr\vert K_{\frac{1}{4}}(\alpha)\,d\alpha \\& \quad \ll N^{-5\delta }\max \bigl\vert F_{1}(\lambda_{1} \alpha)\bigr\vert ^{\frac{1}{5}} \biggl( \int_{-\infty }^{+\infty }\bigl\vert F_{1}( \lambda_{1}\alpha)\bigr\vert ^{9}\biggr)^{ \frac{9}{32}} \biggl( \int_{-\infty }^{+\infty }\bigl\vert F_{2}( \lambda_{2}\alpha)\bigr\vert ^{9}\biggr)^{ \frac{3}{4}} \\& \qquad {} \times \Biggl(\prod_{j=3}^{5} \int_{-\infty }^{+\infty }\bigl\vert F_{j}( \lambda_{j}\alpha)\bigr\vert ^{16} K_{\frac{1}{2}}( \alpha)\,d\alpha \Biggr)^{ \frac{1}{16}} \Biggl(\prod_{k=6}^{8} \int_{-\infty }^{+\infty } \bigl\vert F_{k}( \lambda_{k}\alpha)\bigr\vert ^{32}K_{\frac{1}{2}}(\alpha)\,d\alpha \Biggr) ^{ \frac{1}{32}} \\& \qquad {}\times \biggl( \int_{-\infty }^{+\infty }\bigl\vert G(-\alpha)\bigr\vert ^{2}K_{\frac{1}{2}}( \alpha)\,d\alpha \biggr)^{\frac{1}{2}} \\& \quad \ll X^{\frac{121}{60}-6\delta +\varepsilon } \end{aligned}$$

from Lemmas 5.1, 6.1 and Schwarz’s inequality. □

## Results

In this paper, we established that if $\lambda_{1},\lambda_{2},\ldots,\lambda_{8}$ are positive real numbers, at least one of the ratios $\lambda_{i}/\lambda_{j}$ ($1\leq i< j\leq 8$) is irrational, then the integer parts of $\lambda_{1}x_{1}^{3}+\lambda_{2}x_{2}^{3}+\lambda _{3}x_{3}^{4}+\lambda_{4}x_{4}^{5}+\lambda_{5}x_{5}^{6} +\lambda_{6}x _{6}^{7}+\lambda_{7}x_{7}^{8}+\lambda_{8}x_{8}^{5}$ are prime infinitely often for $x_{1},x_{2},\ldots,x_{8}$, where $x_{1},x_{2},\ldots,x _{8}$ are natural numbers.
